# Fascicular Ventricular Tachycardia: An Electrophysiological Paradox

**DOI:** 10.7759/cureus.101647

**Published:** 2026-01-15

**Authors:** Neeraj Joshi, Tom Harris, Harriet Guthrie, Gaurav Joshi, James Rosengarten

**Affiliations:** 1 General Internal Medicine, East Kent Hospitals University NHS Foundation Trust, Margate, GBR; 2 Cardiology, East Kent Hospitals University NHS Foundation Trust, Margate, GBR; 3 Internal Medicine, East Kent Hospitals University NHS Foundation Trust, Margate, GBR; 4 General Practice, University Institute of Pharma Sciences, Chandigarh University, Mohali, IND

**Keywords:** antiarrhythmic therapy, belhassen ventricular tachycardia, catheter ablation, fascicular ventricular tachycardia, recurrent ventricular tachycardia, ventricular tachycardia, verapamil-sensitive ventricular tachycardia

## Abstract

We present a case of fascicular ventricular tachycardia (VT) in a 27-year-old man. Fascicular VT arises within the fascicles of the left bundle of His and is associated with right bundle branch block and axis deviation on ECG. Fascicular VT can often be mislabelled as a narrow-complex tachycardia due to the proximity of the arrhythmic origin to the normal conduction system, creating a widened, but not classically “broad,” QRS complex, as occurred in our case. Our patient was resistant to adenosine but sensitive to verapamil, as is characteristic of fascicular VT owing to its origin in a macroscopic reentry circuit within the left bundle of His. Indeed, the patient developed recurrences of fascicular VT whenever verapamil doses were missed. Due to the potential for future hemodynamic instability and myocardial ischemia associated with VT, ablation was considered as a long-term management option for this patient. However, because of his high BMI and the associated theoretical reduction in efficacy, ablation was delayed until the patient achieved weight loss. Further research is needed to determine whether obesity affects ablation outcomes in fascicular VT.

## Introduction

Within clinical practice, broad complex tachycardias are typically attributable to ventricular tachycardia (VT) [1]. VTs occur largely as a consequence of structural heart disease, for example, myocardial infarction [2]. However, VT need not occur exclusively in structurally abnormal hearts. Within the spectrum of VTs in structurally normal hearts, approximately 15% originate within the fascicular system of the bundle of His [3]. Typically, patients presenting with fascicular VT are between the ages of 15 and 40 and are predominantly male [4]. Difficulty in identifying these fascicular VTs through ECG analysis can arise, however, since the responsible reentrant circuit lies close to the native conduction system [5], and thus the QRS complex may not appear as overtly “broad” as in other VT etiologies and instead may be mistaken for a supraventricular tachycardia (SVT), an “electrophysiological paradox.”

Fascicular VT was first described by Cohen et al. in 1972 [6], and in 1981, Belhassen et al. described the responsiveness of fascicular VT to verapamil [7]. These verapamil-sensitive idiopathic left VTs occur primarily as a result of macroscopic reentry conduction circuits within the fascicles of the left bundle of His [5,8,9]. The left bundle branch of His is composed of three fascicles: the left anterior, the left septal (middle), and the left posterior [10]. As such, there are three varieties of fascicular VT, each originating within one of these fascicles. Left anterior fascicular VT is associated with right bundle branch block (RBBB) and right axis deviation on ECG; left septal fascicular VT is associated with a relatively narrow QRS complex and a normal axis; and left posterior fascicular VT is associated with RBBB and left axis deviation (LAD) [4].

In terms of management, fascicular VTs tend to be resistant to adenosine but sensitive to verapamil and catheter ablation [11]. Verapamil is proposed to prolong conduction within the reentry circuit and thus slow the rate of VT [7]. Ablation of the origin of the earliest Purkinje potentials during fascicular VT has also been proposed as a treatment and has demonstrated success in small trials [12,13].

## Case presentation

**Figure 1:**
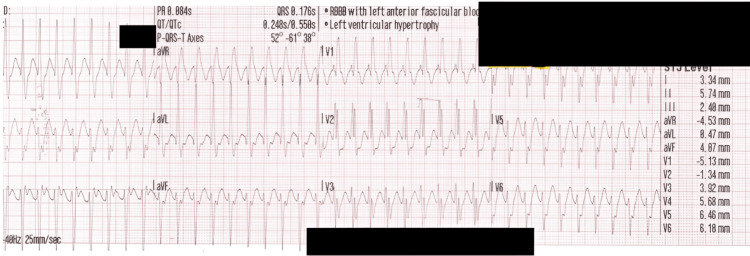
Ambulance ECG from the first admission The ECG shows RBBB and LAD morphology, but with a QRS complex duration of 120-130 ms, leading to diagnostic uncertainty as to whether the complex was broad or narrow. LAD, left axis deviation; RBBB, right bundle branch block

A 27-year-old man with no family history of cardiac disease presented to the emergency department with the sudden onset of palpitations. He had no chest pain, syncope, shortness of breath, or dizziness, and he was hemodynamically stable. The presenting ECG (Figure 1) demonstrated a regular tachycardia with a QRS duration of 120-130 ms, a duration at the threshold (120 ms [14]) between a narrow and broad complex. Subsequently, a diagnosis of SVT was made in the emergency department, the “electrophysiological paradox” discussed in the Introduction.

**Figure 2:**
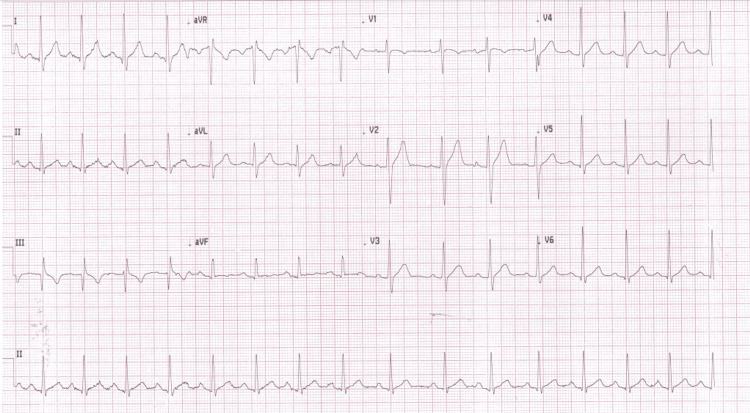
ECG after intravenous verapamil administration The ECG shows a normal sinus rhythm with a normal QRS duration.

Standard acute management for the presumed SVT was commenced. Vagal maneuvers were ineffective, followed by the administration of three escalating doses of adenosine without termination of the arrhythmia. Electrical cardioversion was subsequently attempted, and although sinus rhythm was briefly restored on the third attempt, this was immediately followed by recurrence of the original tachycardia. Intravenous amiodarone 300 mg was administered without effect. Finally, by contrast, administration of intravenous verapamil 10 mg resulted in prompt termination of the tachycardia with restoration of a stable sinus rhythm (Figure 2).

Blood investigations, including electrolytes and thyroid function tests, were within normal limits. High-sensitivity troponin peaked at approximately 500 ng/L without a dynamic rise or fall, and the patient reported no anginal symptoms. The troponin elevation was therefore attributed to tachycardia-mediated myocardial demand rather than acute coronary syndrome. Transthoracic echocardiography demonstrated a structurally normal heart with preserved biventricular systolic function and no significant valvular disease. As the patient remained stable and there was no recurrence of the tachycardia, he was discharged home without medication and with plans for outpatient arrhythmia nurse follow-up. The suspected diagnosis remained an episode of SVT, possibly secondary to an episode of high caffeine intake.

**Figure 3:**
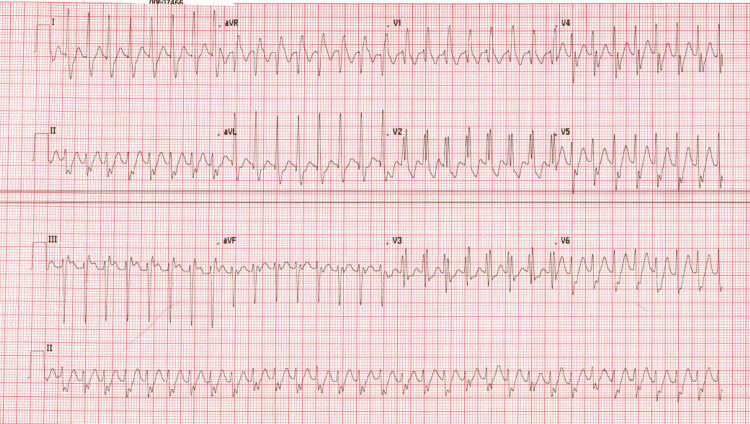
Admission ECG for the second presentation Again, the ECG shows RBBB with LAD, but with an intermediate QRS duration. LAD, left axis deviation; RBBB, right bundle branch block

However, despite significantly reducing his caffeine intake, the patient re-presented six months later with identical symptoms and ECG findings (Figure 3). Once more, the tachycardia failed to respond to typical SVT treatment but subsequently responded promptly to intravenous verapamil. Given the recurrence and failure to respond to adenosine and cardioversion, a cardiology review was sought. Detailed ECG assessment by an electrophysiology consultant identified an RBBB morphology with LAD and a QRS duration of approximately 120-130 ms, again at the borderline between narrow and broad complex [14]. On the basis of the ECG morphology, clinical behavior, and a reproducible response to verapamil, a presumptive diagnosis of fascicular VT was made.

The patient was commenced on regular modified-release verapamil 120 mg once daily. During follow-up, a consistent relationship was observed between missed doses of verapamil and recurrence of palpitations, even in the absence of precipitating factors such as stress or alcohol excess. Indeed, the patient presented a further four times with similar symptoms, and each episode was associated with identical ECG findings and responded reliably to verapamil. This pattern further reinforced the likely diagnosis of fascicular VT. The patient was counseled regarding catheter ablation as a definitive treatment option, although lifestyle modification and weight reduction were advised prior to proceeding with invasive therapy, as he was responding well to oral medication and had not experienced a recurrence in the previous year.

## Discussion

In younger patients with no history of structural heart disease, an important differential diagnosis for broad-complex tachycardia in particular, and for refractory tachycardias in general, remains fascicular VT. In the case discussed above, the patient’s QRS complex was not overtly broad but rather at the borderline between narrow and broad complexes, with a QRS duration of greater than 120 ms as the threshold for a broad complex [14], and the presenting ECG showed a QRS duration of 120-130 ms. However, the pattern of a tachycardia refractory to adenosine and amiodarone but sensitive to verapamil, alongside an ECG showing RBBB and LAD, is consistent with a left posterior fascicular VT. This illustrates the importance of placing ECG findings within the broader clinical context when making diagnoses of tachycardias.

Management of these patients can be complex, given their age and the potential for recurrence. While fascicular VT is sensitive to verapamil and long-term verapamil therapy is often effective at preventing recurrence of VT, as our case demonstrates, the expectation that patients will reliably take verapamil daily for many years can be unrealistic. This leaves open the question of nonpharmacological options to prevent recurrence. As documented in the Introduction, ablation can be a viable alternative. Indeed, Liu et al., in their cohort of 120 patients, found that activation mapping-guided ablation prevented recurrence in all but 15% of patients [13], while Gupta et al. described primary ablation as successful in preventing recurrence in all 108 of their patients [15].

There are drawbacks associated with ablation, however. There are, of course, the risks associated with any invasive procedure, but there are also concerns regarding the efficacy of ablation in patients with increased body mass index. There is limited coverage of the impact of catheter ablation for VTs in the literature. One paper found that obesity was associated with prolonged length of stay and increased risk of pericardial effusions and vascular complications following VT ablation [16]. However, there has been no direct study comparing outcomes in obese versus nonobese patients. As such, most clinical assumptions regarding obesity adversely affecting post-ablation outcomes have been extrapolated from findings in ablation for atrial fibrillation (AF) and flutter.

One systematic review found that for every 5 kg/m² increase in BMI, the risk of AF recurrence increased by 15% following radiofrequency ablation [17], when controlling for other risk factors. Similarly, Ding et al. found that obesity, measured by either waist circumference or BMI, was an independent predictor of AF recurrence (HR 1.25, 95% CI 1.05-1.40) [18]. Further studies have also indicated the utility of weight loss in morbidly obese patients prior to AF ablation in improving ablation outcomes. Indeed, in a meta-analysis of seven cohort studies, Zhao et al. found that every 10% weight loss from baseline significantly reduced the rate of AF recurrence following ablation (RR 0.5, 95% CI 0.33-0.88) [19].

The rationale for the importance of weight loss in improving AF ablation outcomes was applied by the electrophysiologist in our case. Given the patient’s high BMI and the fact that he did not experience any tachycardic events while taking verapamil, with the exception of episodes occurring when he missed doses, it was deemed appropriate to first advise and support weight loss to maximize the prognostic outcome of ablation. While this approach was reasonable in this case, owing to the lack of studies focusing on fascicular VT ablation, we propose that further research is needed to assess the impact of obesity on the efficacy of ablative therapies for fascicular VT before weight loss can be considered a fundamental component of fascicular VT management.

While discussions of fascicular VT can be found in the literature, our case presentation is novel for three reasons. First, our case highlights the previously described “electrophysiological paradox” of fascicular VT: due to the proximity of the arrhythmia origin to the normal conduction pathways, QRS prolongation may be minimal, potentially leading to misdiagnosis as SVT. As such, our case emphasizes the importance of interpreting ECGs within the broader clinical context, for example, a mildly widened QRS complex that is responsive to verapamil being indicative of fascicular VT. Second, our case is unique in discussing the possible role of weight loss in improving the efficacy of ablation for fascicular VT. This relationship has been extrapolated from studies of AF ablation and requires further investigation. Finally, our case is novel in describing a close relationship between adherence to verapamil therapy and recurrence of VT episodes, with a direct correlation between missed doses of verapamil and presentation to the emergency department with VT.

## Conclusions

Fascicular VT is a common cause of idiopathic VT. Diagnosis of this type of VT through ECG analysis can be difficult owing to the proximity of the reentrant circuit to the normal conduction system and the resulting QRS duration at the boundary between SVT and VT. Response to pharmacologic agents can help overcome this diagnostic dilemma, as fascicular VT tends to be resistant to adenosine but sensitive to verapamil. However, these VTs can recur, and in such patients, ablation offers the potential for more definitive management. Preprocedure weight loss may help maximize the efficacy of ablation, but further dedicated research is needed before making a formal recommendation.
